# Effects of peanut consumption on task-related brain activation, cognition and neuroinflammation in older adults with memory complaints: a randomized controlled trial

**DOI:** 10.3389/fnut.2026.1817518

**Published:** 2026-08-21

**Authors:** Gabriela Chiriac, Samuel R. Barnes, Jonathan H. Venezia, Grace J. Lee, Joan Sabate, Sujatha Rajaram

**Affiliations:** 1 School of Public Health, Loma Linda University, Loma Linda, CA, United States; 2 School of Medicine, Loma Linda University, Loma Linda, CA, United States; 3 Department of Psychology, School of Behavioral Health, Loma Linda University, Loma Linda, CA, United States

**Keywords:** age-related cognitive decline, brain activation, cognition, older adults, peanuts

## Abstract

**Objective:**

Peanuts are rich in unsaturated fats, antioxidants like polyphenols, and other bioactive compounds that may reduce neuroinflammation and support cognitive health. Although limited evidence suggests potential cognitive benefits of peanut intake, none have explored these effects in older adults at risk for cognitive decline or assessed brain activity using functional magnetic resonance imaging (fMRI). This study explored the effects of daily peanut consumption on task-related brain activation, cognition, and neuroinflammation biomarkers in older adults with cardiovascular disease (CVD) risk factors and memory complaints.

**Methods:**

In this 12-week, controlled, parallel-design, open-label randomized trial registered at ClinicalTrials.gov (NCT05532007), 30 overweight or obese older adults with memory complaints and CVD risk factors were randomized to a peanut group (PG), which consumed 42–86 g/d of peanuts and peanut butter, or a control group (CG), which abstained from peanuts. The final analyzed sample included 27 participants (PG, *n* = 12; CG, *n* = 15). Assessments at baseline and 12 weeks included fMRI during an n-back working memory task; a comprehensive neurocognitive battery assessing global cognition and specific domains (memory, processing speed, verbal fluency, and executive function); and selected serum markers of inflammation and oxidative stress. Cognitive outcomes were analyzed using analysis of covariance, and biomarker outcomes using repeated-measures models, adjusting for baseline values (cognitive outcomes only), age, sex, baseline BMI, and change in body weight. The Holm step-down procedure was applied separately to cognitive and biomarker outcome families to account for multiple comparisons.

**Results:**

No significant between-group differences were observed in task-related brain activation or cognitive performance. Although some biomarker outcomes were nominally significant in unadjusted analyses, no Time × Group interactions remained significant after adjustment and correction for multiple comparisons.

**Conclusion:**

In overweight or obese older adults with elevated CVD risk and memory complaints, a 12-week peanut intervention did not produce significant changes in cognition or task-related activation of the brain. Future studies with larger sample sizes, longer intervention durations, and greater statistical power are needed to determine whether peanut consumption may influence neurocognitive trajectories and to further investigate the role of inflammatory and oxidative stress mechanisms in these relationships.

## Introduction

Given the global rise in the aging population and the increasing prevalence of neurodegenerative diseases, there is an urgent need for effective, low-cost preventive strategies. Dietary patterns abundant in polyphenols and healthy unsaturated fats, such as the Mediterranean diet, have been linked to neuro- and cardioprotective benefits, including preserved memory and executive function and reduced brain atrophy (1, 2). Evidence from intervention trials with polyphenol-rich foods (e.g., berries, pomegranate, and cocoa) further demonstrates improvements in working memory, processing speed, and brain activity, highlighting the potential for diet to modulate cognition and neural function (3–5).

Peanuts and tree nuts are important dietary sources of polyphenols, antioxidants, vitamin E, niacin, and unsaturated fats, all of which may mitigate oxidative stress and neuroinflammation, key drivers of cognitive decline. Although peanuts contain lower concentrations of polyphenols than anthocyanin-rich foods such as blueberries, they provide a distinct profile of bioactive compounds including proanthocyanidins, phenolic acids, particularly p-coumaric acid, resveratrol, and flavonoids concentrated largely in the peanut skin (6, 7). The roasting process may further modify the polyphenol profile and antioxidant capacity of peanuts, while peanut butter processing disrupts the food matrix and may enhance the bioaccessibility of certain bioactive compounds (6, 8). In addition to these polyphenols, peanuts are rich in monounsaturated fatty acids (MUFA), arginine, and vitamin E, which may act synergistically to improve vascular function, reduce oxidative stress, and support neuronal health (9–15). Therefore, while the bioactive composition of peanuts differs from that of anthocyanin-rich foods, several proposed mechanisms underlying neuroprotection including modulation of inflammation, oxidative stress, endothelial function, and cerebral blood flow may be shared across these dietary interventions.

Although peanuts are widely consumed and nutritionally comparable to tree nuts, their effects on cognition remain largely unexplored. Previous peanut studies reporting cognitive or cerebrovascular benefits have generally used doses of approximately 50–60 g/day (16, 17), while nut interventions demonstrating cardiometabolic and cognitive benefits have typically provided nuts at 15%–20% of daily energy intake (14, 15). Accordingly, the dose selected for this study (42–86 g/day) was designed to provide a physiologically meaningful intake of peanut-derived bioactive compounds while remaining feasible for long-term consumption. Cognitive benefits of nuts and other polyphenol-rich foods may be greatest among individuals at elevated risk for cognitive decline, including those with memory complaints, cardiovascular risk factors, or lower cognitive reserve (14, 18, 19). Moreover, improvements in cognition, cerebral blood flow, and brain activation have been reported following interventions as short as 8–16 weeks (5, 15, 20, 21). However, no studies have examined the effects of peanut consumption on task-related brain activation in overweight and obese older adults at increased risk for cognitive decline. Therefore, this proof-of-concept study investigated the effects of daily peanut consumption on task-related brain activation, cognitive function, insulin resistance, and neuroinflammation in older adults with cardiovascular risk factors and memory complaints.

## Materials and methods

### Study design

This was a 12-week parallel-group, open-label, observer-blind, randomized controlled trial (RCT) (ClinicalTrials.gov NCT05532007) that was retrospectively registered. The primary outcomes were task-related brain activation and cognition, with secondary outcomes including biomarkers of insulin resistance, inflammation, and oxidative stress.

### Study participants

Participants were recruited from Loma Linda, California, and surrounding communities using a multistage recruitment process previously employed by us (22). Following preliminary screening and an informational meeting, interested candidates read and signed the Institutional Review Board approved consent form and were evaluated through clinician interviews before the final selection by the research team. Inclusion criteria were adults aged 60–80 years, with self-reported memory complaints (assessed using a memory complaints questionnaire) and at least two cardiovascular disease (CVD) risk factors: body mass index (BMI) 25–44 kg/m^2^, elevated blood pressure (>130/80 mm Hg), waist circumference >35 inches for women and >40 inches for men, elevated low-density lipoprotein cholesterol (LDL-C) (>130 mg/dL) elevated triglycerides (>150 mg/dL), or use of anti-hypertensive medication. Participants were required to maintain a stable weight for at least 6 months prior to enrollment. Exclusion criteria included dementia, nut allergy, smoking, major metabolic or neurological disorders, chronic disease, claustrophobia (due to MRI), and habitual nut consumption (≥2 servings per week). Sample size was calculated based on an expected moderate effect size (Cohen’s *d* = 0.5) for task-related fMRI activation, informed by the limited nutrition-neuroimaging literature available at the time of study design, including Boespflug et al. (4). Assuming 80% power and α = 0.05, 26 participants were required; allowing for 10% attrition, 30 participants were recruited (15 per group). Given the intensive neuroimaging protocol and comprehensive cognitive assessments, this study was conducted as an exploratory investigation in older adults with CVD risk factors and memory complaints. Observed effect sizes from this trial will provide preliminary estimates to guide sample size calculations for future, adequately powered trials.

### Study protocol and dietary intervention

Participants were randomized 1:1 to the peanut or control group using a computer-generated random number sequence prepared by the study clinician before enrollment. Eligible participants were assigned after completion of screening and baseline assessments. Formal allocation concealment was not used.

Both groups were instructed to maintain their habitual diet throughout the study. Due to the nature of the dietary intervention, participants and personnel involved in intervention delivery were not blinded to group assignment. However, personnel conducting the cognitive assessments and fMRI assessments, as well as personnel performing the biomarker analyses, remained blinded to treatment allocation. Blinding integrity was not formally assessed.

Participants in the peanut group consumed 42–86 g/day of dry-roasted Valencia peanuts with skin and unsalted peanut butter (prepared from roasted peanuts with skin and no other added ingredients), alternating between the two forms every 2 weeks to support adherence. Each participant consumed approximately 6 weeks of whole peanuts and 6 weeks of peanut butter during the 12-week intervention. The peanut butter was prepared from the same peanuts used for the whole-peanut intervention to maintain a comparable nutrient and polyphenol composition across forms, although differences in bioaccessibility between whole peanuts and peanut butter cannot be excluded. The assigned dose provided approximately 20% of estimated daily energy requirements and was consistent with previous peanut intervention studies (15, 16, 23–25).

Control participants excluded peanuts and limited consumption of other nuts to ≤2 servings/week, consistent with our previous nut intervention study (22). No additional dietary advice was provided, and participants were instructed to maintain their habitual diets. The daily peanut dose could be consumed at any time during the day, either in a single serving or divided across multiple occasions. Intervention foods were supplied biweekly, and monthly visits with a study dietitian were used to reinforce adherence.

Estimated energy requirements used to determine peanut dosage were calculated at baseline using a physical activity questionnaire adapted from a validated instrument (26). Participants were instructed to maintain their usual physical activity, medication and supplement use, and other lifestyle habits throughout the study. Adherence and any protocol deviations were reviewed by the study dietitian during monthly clinic visits.

### Data collection

Study outcomes, including task-related brain activation using fMRI, cognitive testing, and blood biomarkers, were assessed at baseline and week 12. Monthly clinic visits were used to provide the intervention to peanut group participants, monitor dietary adherence, and review lifestyle journals.

### Dietary assessment

Dietary intake and adherence were assessed using the Automated Self-Administered 24-Hour Dietary Assessment Tool (ASA24; National Cancer Institute, Bethesda, MD) at baseline and during the intervention period (average of recalls collected at weeks 4, 8, 12). Nutrient intakes were analyzed using the nutrient density method, whereby each nutrient was expressed per 1,800 kcal of total energy intake. The value of 1,800 kcal was selected to approximate the mean energy intake of the study population.

### Functional magnetic resonance imaging

Functional magnetic resonance imaging (fMRI) was performed at baseline and week 12 using a Siemens Skyra 3.0 Tesla scanner with a 32-channel head coil. Participants completed image-based 1-back and 2-back working-memory tasks using a block design consisting of alternating 30-s task and baseline (0-back) conditions. During task blocks, participants viewed a series of images and responded when an image matched that presented one trial earlier (1-back) or two trials earlier (2-back). Each stimulus was presented for 1,000-ms, followed by a 1,000-ms blank interstimulus interval, resulting in a 2,000-ms stimulus onset asynchrony. Participants could respond between 10 and 1,800-ms following stimulus onset. Standardized instructions and a practice session were completed before scanning, and response accuracy was recorded.

Stimuli were presented using Presentation software (v23.0; Neurobehavioral Systems, Inc.). Data were preprocessed using the standard fMRIPrep pipeline (27–30). Preprocessed images were spatially smoothed with a 6-mm full-width-at-half-maximum kernel and intensity-scaled to percent signal change. First-level activation maps were generated in AFNI using a general linear model that included the task design and motion parameters as nuisance regressors.

Whole-brain analyses were conducted using linear mixed-effects models to evaluate voxelwise Time × Group interactions separately for the 1-back and 2-back conditions. Region-of-interest analyses were performed using a Neurosynth meta-analytic working-memory map derived from 1,091 studies and thresholded at false discovery rate (FDR) *q* = 0.01. Mean activation within the region of interest was extracted for each participant and analyzed using the same linear mixed-effects framework applied in the whole-brain analyses.

### Cognitive assessment

The same neurocognitive test battery was administered at baseline and week 12. To minimize practice effects, alternate test forms were used when available. Demographic information (age, sex, and education) was collected at baseline. Cognitive testing was conducted by two trained psychometrists. Participants were randomly assigned to a psychometrist at baseline and assessed by the same psychometrist at week 12 to minimize examiner variability. Standardized instructions were provided before each test. The battery required approximately 45–60 minutes and assessed four cognitive domains:

∙Memory: Rey Auditory Verbal Learning Test (RAVLT) and Brief Visuospatial Memory Test (BVMT).

∙Verbal Fluency: FAS (phonemic fluency) and Animals (semantic fluency).

∙Processing Speed: Symbol Digit Modalities Test (SDMT) and Trail Making Test Part A (TMT-A).

∙Executive Function: Trail Making Test Part B (TMT-B) and Digit Span.

Domain-specific composite scores were calculated by converting individual test scores to z-scores based on the study sample mean and standard deviation and averaging the scores within each cognitive domain. A global cognition score was calculated by averaging z-scores across all cognitive tests.

#### Blood biomarker analyses

Twelve-hour fasting blood samples were collected at baseline and week 12. Serum and plasma were isolated by centrifugation, aliquoted, and stored at −80 °C until analysis at the Human Nutrition Research Center on Aging, Tufts University (Boston, MA). Biomarkers assessed included markers of oxidative stress [8-hydroxy-2′-deoxyguanosine (8-OHdG) and 8-isoprostaglandin F2α (8-iso-PGF2α)], inflammation [tumor necrosis factor-α (TNF-α), interleukin-6 (IL-6), high-sensitivity C-reactive protein (hsCRP), E-selectin, soluble intercellular adhesion molecule-1 (sICAM-1), and soluble vascular cell adhesion molecule-1 (sVCAM-1)], and metabolic health (fasting glucose and insulin). Insulin resistance was estimated using the homeostatic model assessment of insulin resistance (HOMA-IR), calculated as fasting glucose (mg/dL) × fasting insulin (μU/mL)/405 (31). These biomarkers were selected because they reflect pathways related to systemic inflammation, endothelial activation, oxidative stress, and metabolic dysfunction that have been implicated in vascular aging, cognitive decline, and Alzheimer’s disease-related dementia risk (32).

#### Statistical analyses

Statistical analyses were performed using IBM SPSS Statistics version 29.0.1.1 (IBM Corp., Armonk, NY, USA). Analyses were conducted using a complete-case approach. Of the 30 randomized participants, two withdrew before the week-12 assessment and one was excluded after randomization because diabetes, a prespecified exclusion criterion, was identified. The final analytic sample included 27 participants (12 peanut, 15 control), analyzed according to their originally assigned groups.

Assumptions for the parametric analyses were evaluated prior to modeling. For ANCOVA, normality of residuals, linearity, and homogeneity of regression slopes were assessed. Biomarker distributions were examined for normality, and right-skewed variables were log-transformed prior to repeated-measures general linear modeling. Model residuals were inspected to confirm that assumptions were adequately met. Normally distributed variables are presented as means ± standard deviations (SD), whereas adjusted model estimates are presented as estimated marginal means ± standard errors (SE). Non-normally distributed variables are presented as medians and interquartile ranges (IQR).

Cognitive outcomes were analyzed using analysis of covariance (ANCOVA), adjusting for baseline cognitive performance, age, sex, baseline BMI, and change in body weight. Model assumptions were evaluated prior to analysis. Standardized residuals were approximately normally distributed, relationships between cognitive outcomes and continuous covariates were linear, and homogeneity of regression slopes was adequately met for the cognitive composite outcomes.

For fMRI analyses, Group × Time effects were evaluated using linear mixed-effects models in AFNI 3dLME. Whole-brain analyses were conducted using first-level activation maps. For the region-of-interest (ROI) analysis, a Neurosynth meta-analytic association map for the term “working memory” (1,091 studies), thresholded at FDR *q* = 0.01, was registered to MNI152 2009 template space and used to define the ROI (33). Mean ROI activation was analyzed using a linear mixed-effects model with fixed effects for time, group, and the Time × Group interaction.

Because several inflammatory, oxidative stress, and metabolic biomarkers were not normally distributed, within-group changes were examined descriptively using Wilcoxon signed-rank tests. Adjusted intervention effects were evaluated using repeated-measures general linear models on log-transformed biomarker values, with time as the within-subject factor; treatment group and sex as between-subject factors; and age, baseline BMI, and change in body weight as covariates. The primary test of intervention effect was the Time × Group interaction.

To account for multiple comparisons, the Holm step-down procedure was applied separately to the cognitive composite outcomes and biomarker Time × Group interaction tests. Multiplicity in the fMRI analyses was addressed using imaging-specific correction procedures. Both unadjusted and Holm-adjusted *p*-values are reported, and a two-sided Holm-adjusted *p*-value < 0.05 was considered statistically significant.

## Results

### Baseline characteristics

**FIGURE 1:**
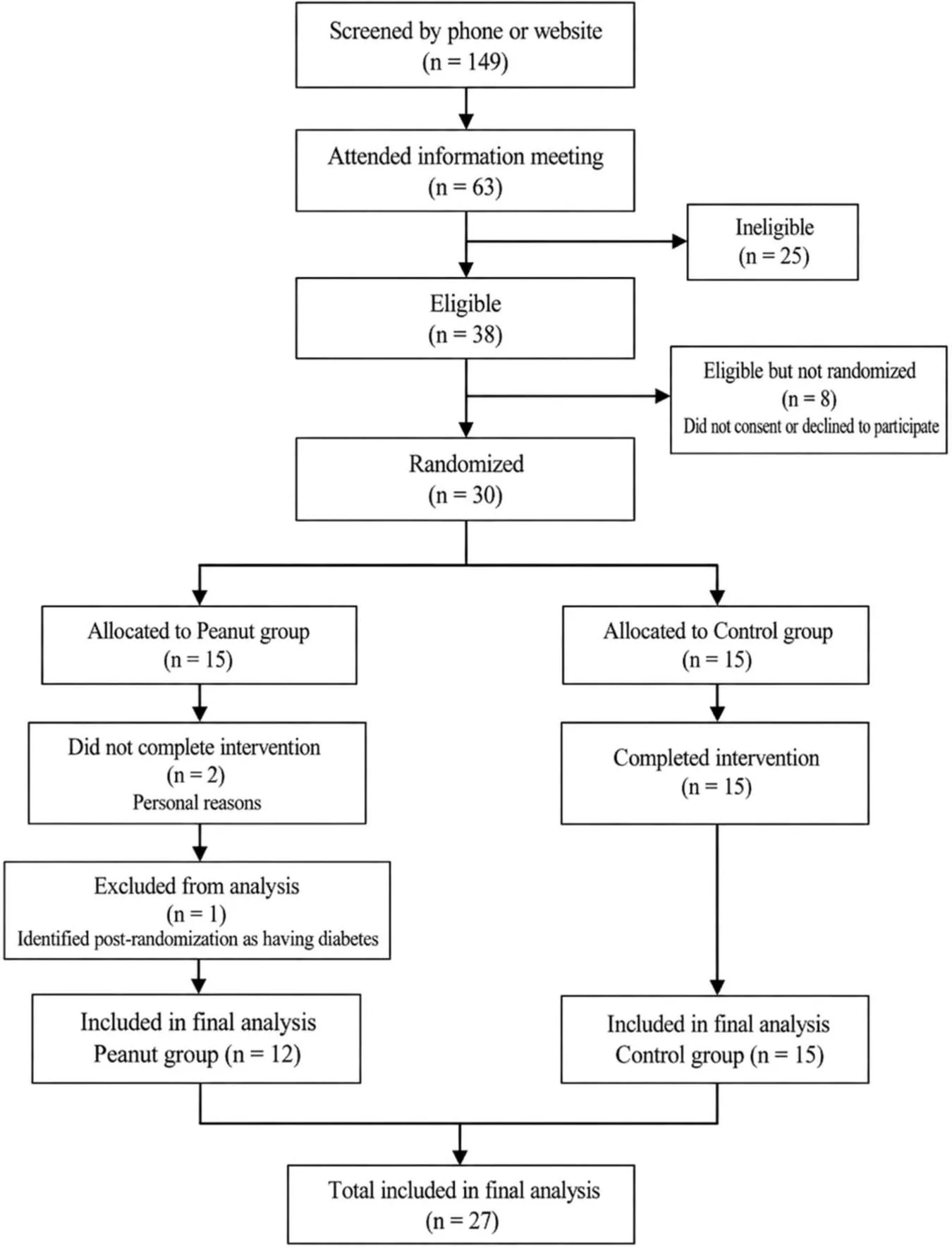
CONSORT flow diagram of participant recruitment, randomization, and retention.

Of the 30 randomized participants (18 women and 12 men), 28 completed the 12-week intervention. Two participants in the peanut group withdrew before the week-12 assessment. One withdrew because caregiving responsibilities for an ill spouse interfered with adherence to the study protocol, and the other withdrew because of concerns about potential weight gain after discussing the intervention with her primary care physician. One additional participant was excluded after randomization because diabetes, a prespecified exclusion criterion, was identified. The final analytic sample included 27 participants (12 peanut, 15 control) (Figure 1).

Participants in the peanut group were significantly older than those in the control group (75.6 ± 4.6 vs. 71.5 ± 5.1 years, respectively; *p* = 0.04). Age was therefore included as a covariate in subsequent analyses. No significant baseline differences were observed in global cognition, the individual cognitive domains, or the other baseline characteristics presented in Table 1.

**TABLE 1:** Baseline characteristics of study participants in the peanut and control groups.

Variables	Peanut group (*n* = 12)	Control group (*n* = 15)	*P*
Age (y)	75.6 ± 4.6	71.5 ± 5.1	0.04*
Education (y)	15.7 ± 2.2	15.0 ± 2.1	0.43
Body weight (kg)	79.6 ± 14.5	84.5 ± 15.6	0.41
BMI (kg/m^2^)	29.1 ± 5.3	30.4 ± 5.5	0.55
Sex, n (%)			
Male	4 (33.3)	6 (40)	0.72
Female	8 (66.7)	9 (60)	
Race/ethnicity, n (%)			
Caucasian	9 (75.0)	8 (53.3)	0.42
Others	3 (25.0)	7 (46.7)	

Values are presented as mean ± standard deviation (SD) for continuous variables and *n* (%) for categorical variables. *P*-values were derived from independent-samples *t*-tests for continuous variables and chi-square tests for categorical variables. *p < 0.05 indicates a statistically significant difference between groups.

Over the 12-week intervention, mean body weight increased by 1.70 ± 1.83 kg in the peanut group and decreased by 0.48 ± 1.57 kg in the control group. The between-group difference in body-weight change was 2.18 kg and was statistically significant (*p* = 0.003).

### Dietary intake

No significant differences in energy-adjusted nutrient intake were observed between groups at baseline (Table 2). During the intervention, the peanut group had higher monounsaturated fat (MUFA) intake and a greater percentage of energy from fat (% energy from fat) than the control group, whereas all other nutrient intakes remained similar between groups.

**TABLE 2:** Energy-adjusted nutrient intake in the peanut and control groups at baseline and during the intervention period.

Nutrients								
	All (*n* = 27)		Peanut group (*n* = 12)		Control group (*n* = 15)		Peanut group VS. Control group^2^	
	Baseline (*n* = 25)^1^	Intervention period (*n* = 27)	Baseline (*n* = 10)^1^	Intervention period (*n* = 12)	Baseline (*n* = 15)	Intervention period (*n* = 15)	Baseline	Intervention period
	Mean (SD)	Mean (SD)	Mean (SD)	Mean (SD)	Mean (SD)	Mean (SD)	*P*-value	*P*-value
Carbohydrate (g)	204.7 (54.1)	209.5 (40.5)	197.5 (42.4)	206.7 (21.5)	209.5 (61.6)	211.8 (51.7)	0.75	0.98
% energy from carbohydrate	46.4 (16)	46.6 (18.5)	43.7 (11.7)	50.9 (22.8)	48.1 (18.5)	43.1 (14.1)	0.72	0.31
Protein (g)	75.7 (23.7)	71.8 (17)	77.3 (16.7)	72 (10.4)	74.7 (27.9)	71.7 (21.3)	0.58	0.72
% energy from protein	16.7 (5.3)	15.6 (5.3)	17.1 (5.3)	17.3 (5.7)	16.4 (5.5)	14.3 (4.7)	0.68	0.14
Total fat (g)	77.7 (20.9)	76.9 (12.4)	81.2 (21.1)	80.8 (9.9)	75.3 (21.2)	73.8 (13.6)	0.49	0.14
% energy from fat	40.7 (18)	37.8 (11.7)	41.5 (15.1)	43 (11.3)	40.1 (20.1)	33.7 (10.6)	0.75	0.04*
Saturated fat (g)	22.6 (8.5)	22.2 (4.4)	22 (7.7)	22.5 (4.4)	23.1 (9.3)	21.9 (4.5)	0.91	0.67
MUFA (g)	29.1 (11.5)	28.6 (6.4)	32 (11.7)	31.8 (4.7)	27.2 (11.4)	26 (6.5)	0.26	0.02*
PUFA (g)	19.2 (6.4)	19.8 (3.9)	20.5 (8)	20.1 (3.8)	18.4 (5.1)	19.6 (4.2)	0.54	0.74
Fiber (g)	22.8 (9.5)	22 (7.8)	21 (9.4)	22.5 (7.6)	24 (9.6)	21.6 (8.3)	0.36	0.75
Cholesterol (mg)	272.9 (202.3)	222.1 (152.5)	272 (188.3)	187.3 (88.1)	273.5 (217.6)	250 (187.7)	0.64	0.59
α-tocopherol (mg)	9.5 (4.2)	10.4 (4.3)	10.4 (5.1)	11.8 (4.8)	8.9 (3.5)	9.2 (3.6)	0.60	0.06

Values are presented as mean ± SD. Baseline values were derived from one dietary recall collected before randomization. Intervention Period values represent the average of dietary recalls collected at weeks 4, 8, and 12. Nutrient intakes were standardized to 1,800 kcal. Between-group comparisons were performed separately at baseline and during the intervention. MUFA, monounsaturated fatty acids; PUFA, polyunsaturated fatty acids.

*P < 0.05 indicates a statistically significant between-group difference.

^1^Indicates that baseline dietary recall data were available for 25 participants overall, including 10 participants in the peanut group, because baseline dietary recall data were unavailable for two peanut-group participants.

^2^Indicates that between-group comparisons were performed separately at baseline and during the intervention period.

Dietary adherence was assessed using ASA24 dietary recalls and records of peanut products dispensed and returned. Among participants in the peanut group, mean peanut intake was 53.4 ± 6.9 g/day, corresponding to 98.6% compliance with the prescribed intake. Mean nut intake in the control group was 5.9 ± 9.0 g/day.

### Functional magnetic resonance imagining

**FIGURE 2:**
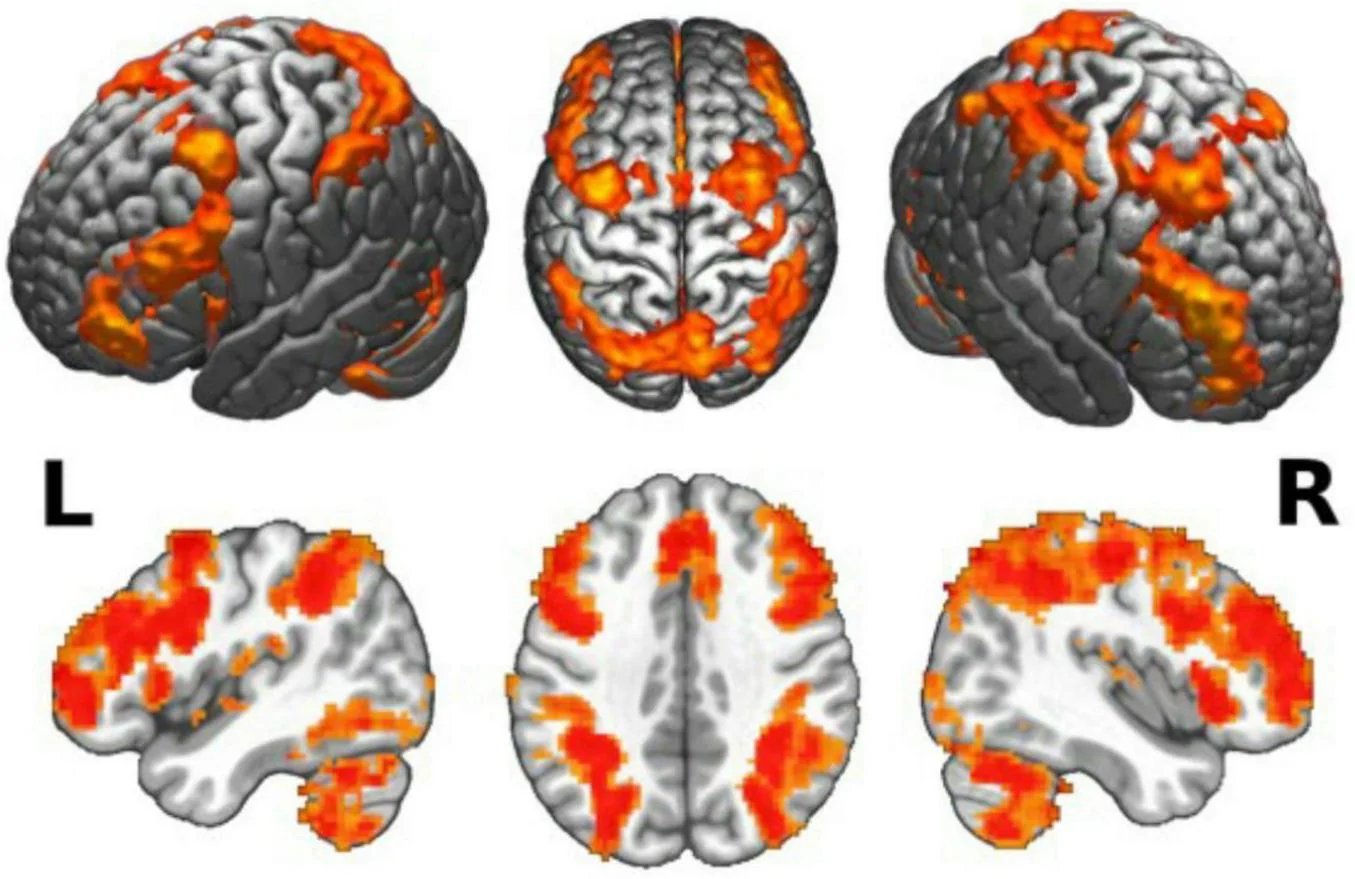
Whole-brain task-related activation during the 2-back working memory task for both groups for baseline and week 12. Statistical parametric map of the mean task-versus-baseline (2-back vs. 0-back) BOLD response across all participants and both time points (baseline and week 12), estimated with the group-level linear mixed-effects model (AFNI 3dLME). Maps are thresholded at FDR *q* < 0.001 and displayed on lateral, medial, and superior cortical surface views. The color bar represents the activation F-statistic; warmer colors indicate greater task-related activation. L = left hemisphere; R = right hemisphere.

**FIGURE 3:**
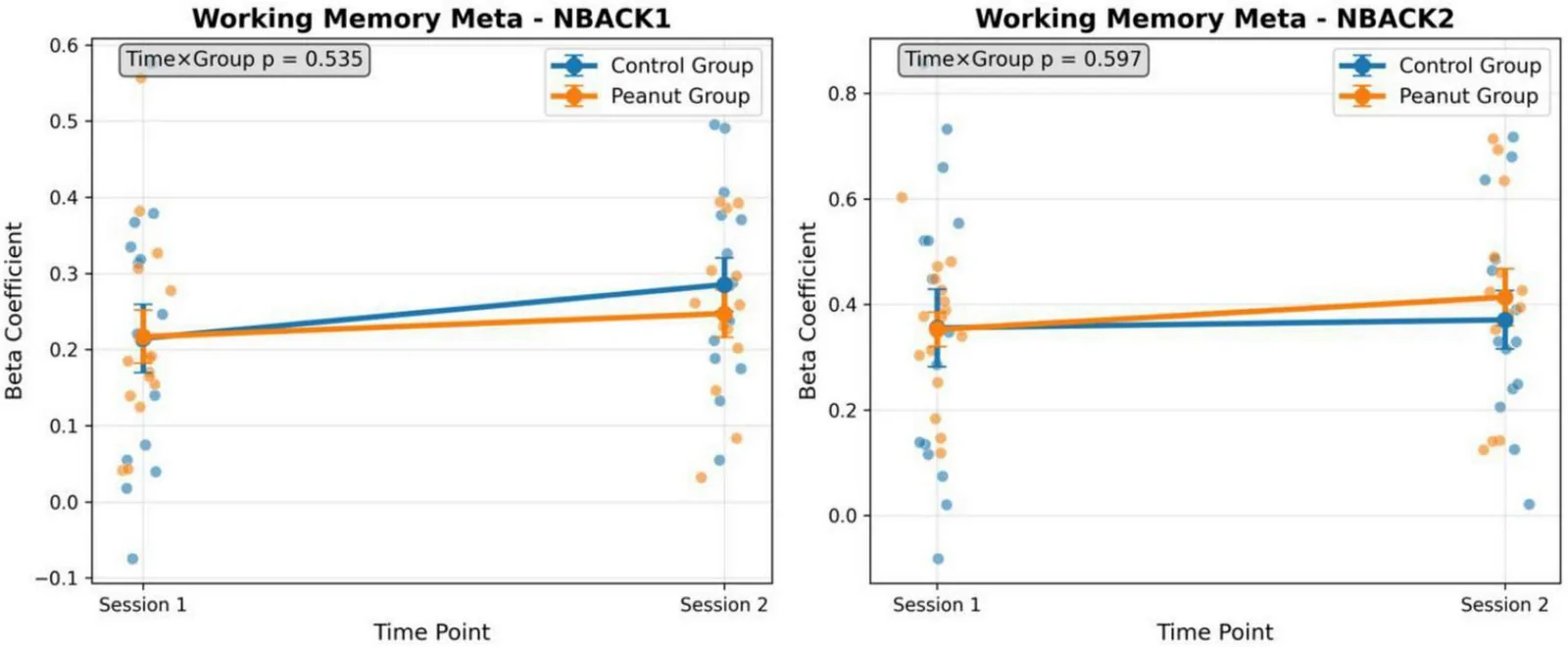
Task-related brain activation during the working memory fMRI task (1-Back and 2-Back): changes from baseline to week 12 in peanut and control groups. Mean working-memory ROI activation (first-level beta coefficient) from baseline to week 12 in the peanut (orange; *n* = 12) and control (blue; *n* = 15) groups, for the 1-back and 2-back conditions. Points are individual participants; error bars denote standard error of the mean. Group differences in change over time were tested with a linear mixed-effects model (Time × Group interaction): 1-back *p* = 0.535; 2-back *p* = 0.597. ROI definition and model details are given in the section “Materials and methods.”

Analysis of the 2-back fMRI data across all participants and time points showed expected activations in prefrontal, parietal, and temporal regions, confirming engagement of working memory networks (Figure 2). Linear mixed-effects modeling did not reveal statistically significant group x time interactions; however, patterns of activations suggest potential trends in working memory regions that may warrant exploration in larger studies. The working memory ROI analysis also did not detect any group x time interactions (Figure 3) for 1-back (*p* = 0.535) or 2-back (*p* = 0.597).

### Cognitive function

Baseline cognitive performance was similar between groups across all domains (Table 3). No significant between-group differences were observed for global cognition or any domain-specific cognitive composite after adjustment for covariates (Table 4). Processing speed showed the largest numerical between-group difference, with greater improvement in the control group than in the peanut group; however, this difference was not statistically significant before or after correction for multiple comparisons (unadjusted *p* = 0.067; Holm-adjusted *p* = 0.335).

**TABLE 3:** Baseline cognitive performance in the peanut and control groups.

Cognitive domain	Cognitive measure	Peanut group mean ± SD	Control group mean ± SD	*P*-value
Memory	Composite	−0.05 ± 0.75	0.04 ± 0.87	0.75
	RAVLT immediate	41.08 ± 9.20	39.80 ± 11.54	0.75
	RAVLT delayed	7.83 ± 2.36	7.80 ± 3.38	0.97
	BVMT immediate	14.50 ± 6.15	16.87 ± 6.26	0.33
	BVMT delayed	6.42 ± 2.76	6.80 ± 2.48	0.7
Verbal fluency	Composite	−0.22 ± 1.02	0.15 ± 0.82	0.29
	FAS	40.67 ± 12.19	43.13 ± 13.09	0.62
	Animals	17.25 ± 5.64	19.87 ± 3.77	0.16
Processing speed	Composite	−0.19 ± 0.78	0.17 ± 0.82	0.25
	SDMT	39.67 ± 7.13	41.4 ± 6.74	0.52
	TMT-A	28.69 ± 8.48	24.86 ± 7.77	0.23
Executive function	Composite	−0.13 ± 0.81	0.11 ± 0.69	0.41
	TMT-B	91.32 ± 36.95	70.06 ± 26.33	0.093
	Digit span	25.75 ± 5.05	25 ± 4.97	0.7
Global cognition	Composite	−0.12 ± 0.61	0.10 ± 0.52	0.28

Values are presented as unadjusted means ± SD. Composite scores represent the standardized z-scores derived from the individual tests within each cognitive domain. Baseline between-group comparisons were performed using independent-samples *t*-tests. RAVLT, Rey Auditory Verbal Learning Test; BVMT, Brief Visuospatial Memory Test; SDMT, Symbol Digit Modalities Test; TMT-A, Trail Making Test Part A; TMT-B, Trail Making Test Part B.

**TABLE 4:** Adjusted cognitive composite scores at baseline and week 12 in the peanut and control groups.

Cognitive composite	Peanut group adjusted mean ± SE	Control group adjusted mean ± SE	*P*
Memory			
Baseline	−0.080 ± 0.264	0.059 ± 0.234	
Week 12	0.079 ± 0.213	0.248 ± 0.188	
Adjusted change	0.160 ± 0.167	0.189 ± 0.148	0.9
Verbal fluency			
Baseline	−0.292 ± 0.234	0.212 ± 0.207	
Week 12	−0.001 ± 0.206	0.345 ± 0.183	
Adjusted change	0.291 ± 0.163	0.132 ± 0.145	0.49
Processing speed			
Baseline	−0.273 ± 0.248	0.234 ± 0.219	
Week 12	−0.316 ± 0.266	0.583 ± 0.236	
Adjusted change	−0.043 ± 0.146	0.349 ± 0.129	0.067
Executive function			
Baseline	−0.121 ± 0.216	0.106 ± 0.191	
Week 12	0.065 ± 0.168	0.281 ± 0.149	
Adjusted change	0.186 ± 0.155	0.176 ± 0.137	0.96
Global cognition			
Baseline	−0.169 ± 0.177	0.134 ± 0.156	
Week 12	−0.019 ± 0.169	0.341 ± 0.149	
Adjusted change	0.151 ± 0.088	0.207 ± 0.078	0.65

Values are presented as estimated marginal means ± standard errors (SE). Baseline and week 12 values were derived from adjusted models including age, sex, baseline BMI, and change in body weight as covariates. Adjusted change values represent the estimated change from baseline to week 12. *P*-values represent between-group comparisons of adjusted change scores derived from analysis of covariance models. To account for multiple comparisons across the five cognitive composite outcomes, the Holm step-down procedure was applied. No cognitive outcome remained statistically significant after Holm correction; the smallest adjusted *p*-value was observed for processing speed (Holm-adjusted *p* = 0.335).

### Inflammatory and oxidative stress markers

Within-group analyses showed reductions in IL-6 in both the peanut (*p* = 0.026) and control (*p* = 0.043) groups, whereas no significant within-group changes were observed for the remaining inflammatory and oxidative stress biomarkers (Table 5). In covariate-adjusted repeated-measures analyses, no inflammatory or oxidative stress biomarker demonstrated a significant Time × Group interaction after Holm correction for multiple comparisons (Table 5). The smallest unadjusted *p*-value was observed for 8-OHdG [*F*(1, 21) = 3.825, *p* = 0.064, partial η^2^ = 0.154], which remained non-significant after Holm correction (adjusted *p* = 0.640).

**TABLE 5:** Baseline and week 12 inflammatory and oxidative stress biomarkers by intervention group and Time × Group interaction analyses.

Biomarker	Peanut baseline median (IQR)^†^	Peanut week 12 median (IQR)^†^	Control baseline median (IQR)^†^	Control week 12 median (IQR)^†^	*F* (1,21)	Raw *p*	Holm-adjusted *p*
Glucose (mg/dL)	97.50 (88.75–113.50)	102.50 (95.25–116.25)	98.00 (94.00–103.00)	97.00 (92.00–105.00)	0.123	0.729	1.000
Insulin (μIU/mL)	13.90 (8.27–18.67)	14.45 (8.37–21.60)	8.50 (7.30–13.20)	9.40 (7.30–14.20)	1.376	0.254	1.000
HOMA-IR	3.58 (1.87–4.71)	3.31 (1.93–6.20)	2.09 (1.71–3.03)	2.08 (1.82–3.71)	1.038	0.320	1.000
TNF-α (pg/mL)	0.94 (0.83–1.22)	1.04 (0.79–1.32)	0.97 (0.84–1.08)	0.86 (0.76–1.14)	0.007	0.936	1.000
IL-6 (pg/mL)	0.62 (0.47–0.82)	0.46 (0.34–0.63)	0.58 (0.33–0.90)	0.51 (0.31–0.63)	0.326	0.574	1.000
hsCRP (mg/L)	2.80 (1.10–5.37)	2.00 (0.95–3.95)	2.80 (0.50–7.30)	1.90 (1.10–5.30)	0.534	0.473	1.000
E-selectin (ng/mL)	26.45 (24.35–37.22)	27.30 (22.12–27.30)	29.30 (24.30–38.30)	33.40 (26.90–41.10)	2.077	0.164	1.000
sICAM-1 (ng/mL)	363.50 (324.00–447.00)	372.50 (316.25–452.50)	404.00 (325.00–517.00)	413.00 (353.00–481.00)	5.213	0.033	0.363
sVCAM-1 (ng/mL)	484.50 (415.50–632.75)	491.50 (426.00–613.50)	578.00 (521.00–617.00)	568.00 (537.00–635.00)	6.017	0.023	0.276
8-OHdG (ng/mL)	28.05 (18.85–35.17)	28.75 (20.97–37.30)	27.90 (25.10–34.10)	26.10 (22.90–31.90)	3.825	0.064	0.640
8-iso-PGF_2_α (pg/mL)	59.15 (54.40–84.12)	68.60 (57.42–74.15)	71.30 (57.10–76.20)	60.10 (49.00–70.30)	0.282	0.601	1.000
MMA (pmol/mL)	200.00 (136.50–290.75)	212.00 (177.75–366.50)	229.00 (144.00–255.00)	214.00 (132.00–292.00)	1.396	0.251	1.000

hsCRP, high-sensitivity C-reactive protein; HOMA-IR, homeostatic model assessment of insulin resistance; TNF-α, tumor necrosis factor-α; IL-6, interleukin-6; sICAM-1, soluble intercellular adhesion molecule-1; sVCAM-1, soluble vascular cell adhesion molecule-1; 8-OHdG, 8-hydroxy-2′-deoxyguanosine; 8-iso-PGF2α, 8-isoprostaglandin F2α; MMA, methylmalonic acid.

†Values are presented as median (interquartile range [IQR; 25th–75th percentile]). Repeated-measures general linear models were performed on log-transformed biomarker values. F statistics and *p*-values represent the Time × Group interaction, adjusted for age, sex, baseline BMI, and change in body weight. Raw interaction *p*-values were corrected for multiple comparisons using the Holm step-down procedure across the 12 biomarkers.

### Metabolic markers

No significant within-group changes were observed in fasting glucose, insulin, HOMA-IR, or methylmalonic acid concentrations over the 12-week intervention (Table 5). Similarly, covariate-adjusted repeated-measures analyses showed no significant Time × Group interactions for any metabolic marker before or after correction for multiple comparisons (Table 5).

Across all biomarker outcomes, no Time × Group interaction remained statistically significant after Holm correction for multiple comparisons (Table 5). The smallest unadjusted *p*-values were observed for sVCAM-1 (*p* = 0.023), sICAM-1 (*p* = 0.033), and 8-OHdG (*p* = 0.064), but none remained significant after adjustment.

## Discussion

In this exploratory trial, daily peanut consumption did not yield statistically significant improvements in cognitive performance, brain activation, or inflammatory markers. These results nevertheless provide valuable insights into factors that may influence neural and cognitive responsiveness to dietary interventions in older adults with elevated adiposity and cardiovascular risk factors. The limited responsiveness observed may reflect the metabolic and adiposity profile of the participants, including overweight and obesity, low-grade chronic inflammation, and insulin resistance, which are known to attenuate the physiological and neurocognitive effects of peanuts and polyphenol-rich foods (15, 16, 34, 35). These findings underscore the importance of considering baseline metabolic status, intervention dose, and duration when evaluating the potential cognitive benefits of peanuts and other nutrient-dense foods in at-risk older populations and provide preliminary effect size estimates to guide the design of future, adequately powered trials.

Previous research has reported cognitive benefits of long-term nut consumption. In older adults, a Mediterranean diet (2) supplemented with mixed nuts improved memory and executive function after nearly 5 years of intervention. Similarly, in the Walnut and Healthy Aging (WAHA) trial we observed that 2 years of eating walnuts daily improved global cognition and perception scores, though the benefits were limited to individuals with lower cognitive reserves (e.g., lower education level or reduced red blood cell (RBC) α-linolenic acid levels) (14). While these findings emphasize the value of long-term exposure, shorter interventions of 12 weeks or longer have demonstrated meaningful effects on cognition and brain activation. Studies with blueberries, pomegranate juice, and peanuts have shown improvements in memory, verbal fluency, and brain activity within this timeframe, suggesting that relatively brief dietary intervention may be sufficient to elicit neurocognitive benefits in certain populations (18, 36). Thus, factors other than intervention duration, including participant adiposity and metabolic status, may warrant consideration as potential modifiers of responsiveness to dietary interventions.

We previously reported that the LDL-C–lowering effects of nuts are attenuated in individuals with obesity (37), suggesting that excess adiposity may influence responsiveness to nut-derived bioactive compounds. Similarly, some studies of polyphenol-rich foods have reported weaker cognitive and neurovascular responses in overweight and obese populations (3, 4, 22, 36). In the present study, all participants were overweight or obese and exhibited evidence of chronic low-grade inflammation and insulin resistance at baseline. Although not directly tested, these metabolic and inflammatory characteristics may have contributed to the limited responsiveness observed. While age is an important determinant of cognitive function, future studies should examine whether baseline adiposity, inflammation, and metabolic health help explain variability in response to dietary interventions.

In addition to participant characteristics, methodological factors related to neuroimaging assessment may also have contributed to the absence of detectable intervention effects. Although a recent peanut intervention reported improvements in cerebral blood flow after 16 weeks (18), to our knowledge this is the first study to evaluate task-related brain activation in response to peanut consumption. We did not observe changes in brain activation, whereas studies of other polyphenol-rich foods, particularly blueberries, have reported enhanced activation during cognitive tasks (4, 20). For example, Boespflug et al. (4) reported improvements in working memory performance accompanied by increased activation in the left precentral gyrus and inferior parietal lobe, particularly during higher cognitive load conditions (2-back tasks).

Several methodological differences may help explain these discrepant findings. Boespflug et al. (4) employed a sequential letter-based working memory task, whereas our study used image-based stimuli that require additional visual and spatial processing. These added demands may engage broader neural networks and reduce task-specific activation within canonical working memory regions (38). Differences in task selection across nutrition intervention studies may also contribute to variability in fMRI outcomes. For example, pomegranate interventions have used visual, verbal, and spatial memory tasks emphasizing memory retrieval and learning (5), whereas blueberry studies have employed numerical Stroop tasks that primarily assess attention, processing speed, and inhibitory control (20). In contrast, the n-back paradigm used in the present study is considered a sensitive measure of working memory function and its associated neural circuitry (39–41). Collectively, these methodological differences highlight how task selection and stimulus complexity may influence observed neural responses and contribute to variability across nutrition intervention studies.

Estimated polyphenol intake from the peanut intervention ranged from approximately 48 to 97 mg/day, based on polyphenol contents reported in the Phenol-Explorer database (7) and the prescribed quantities of peanuts and peanut butter. This exposure was substantially lower than the approximately 387–417 mg/day provided in blueberry interventions that have reported cognitive benefits (20), which may partly explain the absence of detectable effects on cognition and brain activation. Future studies should evaluate whether alternative peanut formulations or higher doses can enhance bioactive compound delivery and improve the likelihood of detecting neurocognitive effects.

Although no overall cognitive benefits were observed, processing speed showed the largest numerical between-group difference and therefore warrants brief consideration. Greater improvement was observed in the control group than in the peanut group; however, the effect was not statistically significant before or after correction for multiple comparisons. Because processing speed was assessed using repeated cognitive tests, some improvement may reflect practice effects despite the use of alternate test forms when available. Chance variation, test–retest variability, regression to the mean, or residual effects of baseline group differences may also have contributed to the observed pattern. Importantly, this finding was not accompanied by differences in global cognition, other cognitive domains, or task-related brain activation. Accordingly, the result should be interpreted cautiously given the exploratory nature of the study and modest sample size.

Consistent with previous studies of peanuts and tree nuts, peanut consumption had limited effects on inflammatory, oxidative stress, and metabolic biomarkers despite participants being overweight and exhibiting evidence of chronic low-grade inflammation. Although IL-6 decreased over time in both groups, the absence of a significant Time × Group interaction suggests that this change was not specific to the intervention. Similarly, nominal reductions in endothelial activation markers, including sVCAM-1, did not remain significant after correction for multiple comparisons.

These findings are consistent with shorter-duration peanut interventions that have generally reported minimal effects on inflammatory biomarkers (23, 24, 42). In contrast, longer interventions such as the WAHA study reported reductions in IL-6, TNF-α, and E-selectin following 2 years of walnut consumption (43). Differences in intervention duration, nut type, and participant characteristics may partly explain these discrepant findings. Because obesity is associated with chronic inflammation, oxidative stress, and metabolic dysfunction, it remains plausible that these factors influence responsiveness to dietary interventions; however, this hypothesis was not directly evaluated in the present study.

A major strength of this study was the comprehensive assessment of neurocognitive function, including domain-specific cognitive composites and task-related fMRI, which provided complementary measures of cognitive performance and neural activity in a population at elevated risk for cognitive decline. The high level of dietary adherence further supports the validity of the intervention and outcome assessments.

Several limitations should be acknowledged. First, this was an exploratory study with an open-label design. Sample size calculations were based on the primary fMRI outcome using effect estimates available at the time of study design. Consequently, the study may have been underpowered to detect smaller but potentially meaningful effects on secondary outcomes, including cognitive performance and circulating biomarkers. Therefore, null findings for these outcomes should be interpreted with caution.

Participants in the peanut group were older than those in the control group at baseline; although age was included as a covariate and baseline cognitive performance did not differ between groups, residual confounding cannot be excluded. Second, despite instructions to maintain stable body weight, the peanut group gained weight whereas the control group lost weight. Although change in body weight was included as a covariate in the cognitive and biomarker analyses, residual effects of differential weight change may have influenced the findings. Third, the 12-week intervention may have been insufficient to detect meaningful changes in cognition, brain activation, or circulating biomarkers. Fourth, although alternate cognitive test forms were used when available and multiple-comparison correction was applied, the large number of outcomes examined and limited sample size reduced statistical power and increased the likelihood of both false-negative and chance findings. Finally, sample size calculations were based on the primary fMRI outcome; therefore, the study may have been underpowered to detect smaller but potentially meaningful effects on secondary cognitive and biomarker outcomes. Consequently, the findings may not be generalizable beyond overweight or obese older adults with cardiovascular risk factors and memory complaints.

This study is among the first to investigate the effects of peanut consumption on cognitive performance, task-related brain activation, and neuroinflammatory biomarkers in older adults with memory complaints and cardiovascular risk factors. Although the 12-week intervention did not produce significant improvements in cognitive, neuroimaging, or biomarker outcomes, the findings provide important information regarding the feasibility of dietary interventions targeting neurocognitive health in at-risk populations and help identify factors that may influence responsiveness, including intervention duration, bioactive exposure, and underlying metabolic status. Future studies should evaluate longer interventions, alternative peanut formulations, and populations with greater potential responsiveness to determine whether peanut consumption can contribute to the maintenance of cognitive health during aging.

## Data Availability

The raw data supporting the conclusions of this article will be made available by the authors, without undue reservation.
